# Enveloped Ablation: A Strategy for Managing Outflow Tract PVCs With an R Wave Pattern Break in Lead V2

**DOI:** 10.1155/cric/4667457

**Published:** 2025-11-14

**Authors:** Jiro Koya, Taro Temma, Motoki Nakao, Masaya Watanabe, Toshihisa Anzai

**Affiliations:** Department of Cardiovascular Medicine, Faculty of Medicine and Graduate School of Medicine, Hokkaido University, Sapporo, Japan

**Keywords:** low-power, long-duration ablation, outflow tract arrhythmias, premature ventricular contraction, R wave pattern break in Lead V2 (PBV2), radiofrequency catheter ablation

## Abstract

Outflow tract premature ventricular contractions (OT-PVCs) with an R wave pattern break in Lead V2 (PBV2) pose significant treatment challenges due to their refractory nature and complex anatomical origins. A 56-year-old male with drug-resistant palpitations underwent detailed electroanatomical mapping using a microcatheter to identify the earliest activation site. This precision mapping was crucial for directing the ablation strategy accurately. The “enveloped ablation” technique was employed, involving multisite, low-power ablations surrounding the critical activation site, tailored to address the unique electrical and structural characteristics of OT-PVCs with a PBV2. This case highlights the importance of accurate mapping and tailored ablation strategies in managing OT-PVCs with PBV2.

## 1. Introduction

Ablation therapy for outflow tract premature ventricular contractions (OT-PVCs) has become a well-established definitive treatment [1, 2]. However, OT-PVCs characterized by an R wave pattern break in Lead V2 (PBV2), where the R wave amplitude in Lead V2 is smaller than in Leads V1 and V3, are notably refractory and lack a standardized treatment strategy [3, 4]. We present a case in which detailed electroanatomical mapping and an innovative multisite ablation approach surrounding the early activation site successfully treated OT-PVCs with a PBV2.

## 2. Case Presentation

A 56-year-old male with a 5-year history of palpitations (body mass index: 20.2 kg/m^2^) was diagnosed with PVCs on Holter monitoring. Despite treatment with beta-blockers, the frequency of PVCs remained unchanged. Due to drug resistance, the patient underwent catheter ablation.

**Figure 1:**
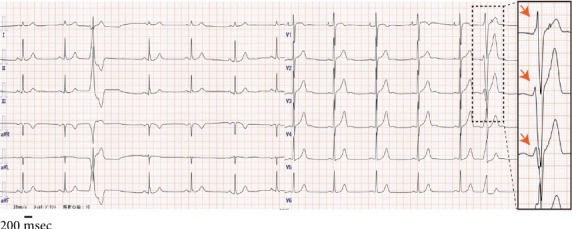
Electrocardiogram. Premature ventricular contractions exhibiting an inferior axis, left bundle branch block type morphology, and transition zone in V3–V4. An enlarged view of Leads V1–V3 is provided for clarity. A marked reduction in the R wave amplitude was noted in Lead V2, compared with Leads V1 and V3, and is highlighted with red arrows to illustrate the pattern break.

The 12-lead ECG findings revealed PVCs with an inferior axis and left bundle branch block morphology, with a transition zone in Leads V3 and V4. A noticeable R wave decrease in Lead V2, compared to Leads V1 and V3, led to a diagnosis of OT-PVCs with a PBV2 (Figure 1). The correct placement of precordial leads was carefully verified at the time of recording. A Holter ECG recorded a PVC burden of 17.2% per day.

**Figure 2:**
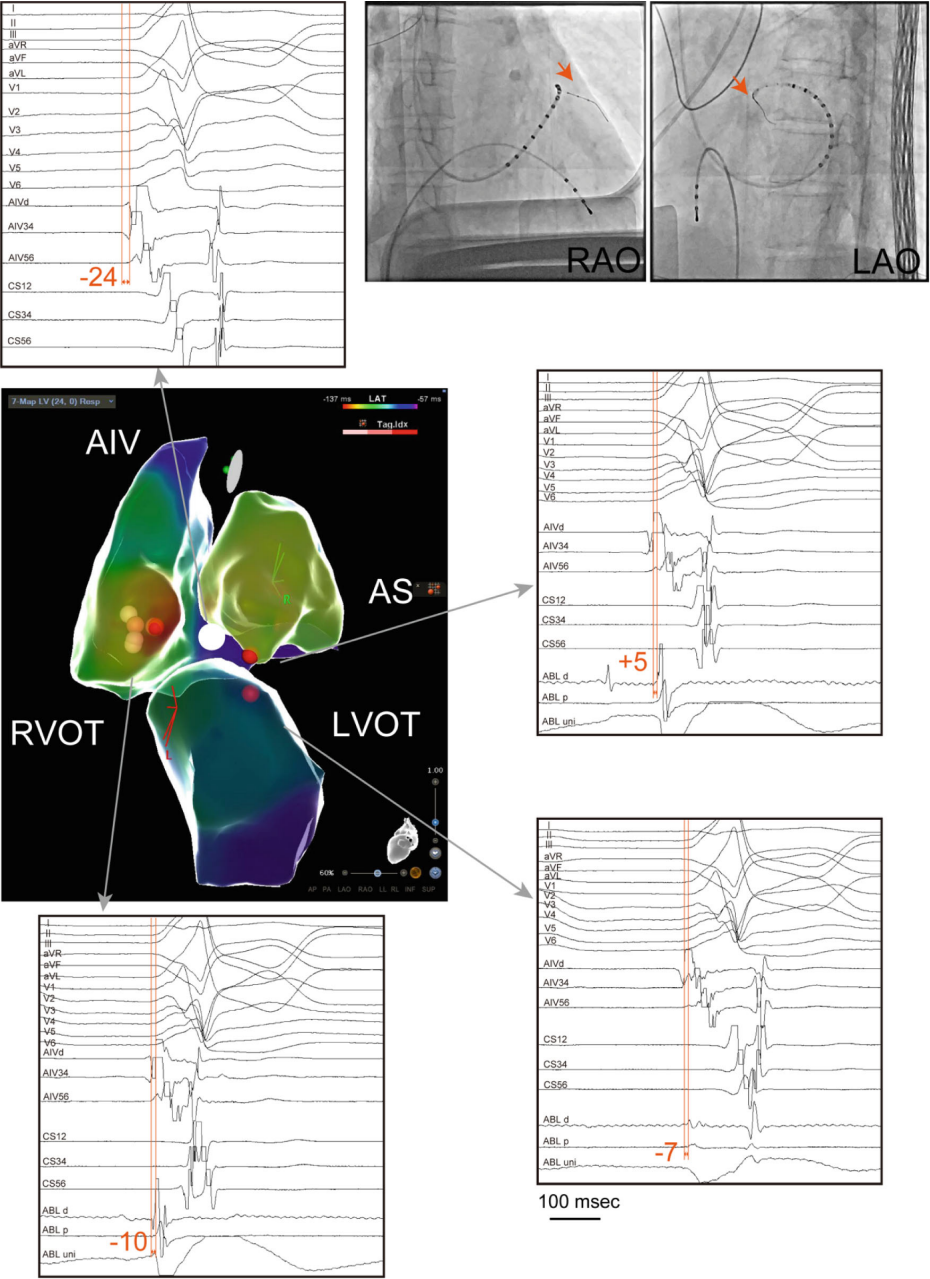
Ablation catheter electrogram and activation mapping. A 2.6-Fr electrode catheter was placed in the anterior interventricular vein (AIV), as indicated by the orange arrows. The AIV region demonstrated the earliest activation relative to the onset of the QRS complex, occurring at −24 ms. Detailed mapping of the right ventricular outflow tract (RVOT), left ventricular outflow tract (LVOT), and aortic sinus (AS) was conducted using an ablation catheter. The earliest activation site identified by the mapping was in the RVOT (−10 ms), followed by the LVOT (−7 ms) and AS (+5 ms). The red tags mark the ablation points. The white tag indicates the earliest PVC site in the AIV.

**Figure 3:**
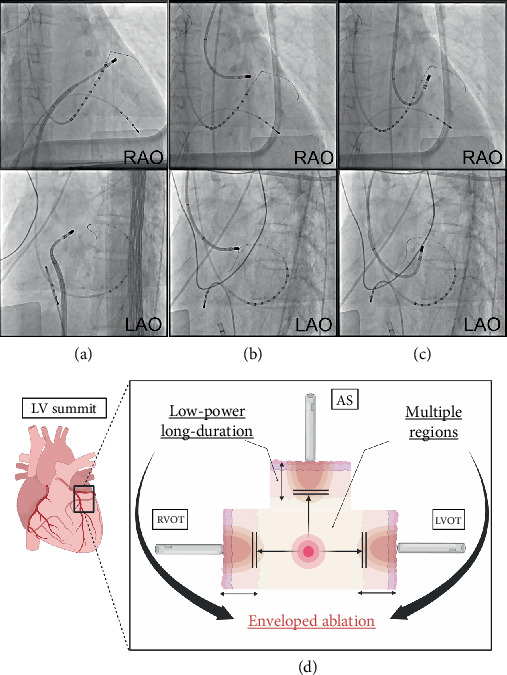
Ablation procedure and graphical schema. Successful ablation sites. (a) First, ablation was started from the right ventricular outflow tract (RVOT) region (20 W for 120 s). (b) Additional ablation was started from the aortic sinus (AS) region (25 W for 65 s). (c) Finally, additional ablation was started from the left ventricular outflow tract (LVOT) region (30 W for 90 s), achieving the suppression of the PVCs, and the procedure was ended. (d) Graphical schema. AS, aortic sinus; LAO, left anterior oblique view; LVOT, left ventricular outflow tract; RAO, right anterior oblique view; RVOT, right ventricular outflow tract.

A 2.6-Fr electrode catheter (EPstar Fix AIV; Japan Lifeline, Tokyo, Japan) was initially inserted into the anterior interventricular vein (AIV). The AIV region revealed an earliest activation in relation to the QRS onset of −24 ms (Figure 2). Importantly, no ablation was performed within the AIV; the catheter was used solely for mapping and as an anatomical landmark. Consequently, detailed mapping of the right ventricular outflow tract (RVOT), left ventricular outflow tract (LVOT), and aortic sinus (AS) was performed using an ablation catheter (THERMOCOOL SMARTTOUCH SF; Biosense Webster, California, United States) in combination with the CARTO 3 three-dimensional electroanatomical mapping system (Biosense Webster, California, United States) to encompass the AIV region. Prior to ablation near the AIV and LV summit, coronary angiography was performed to confirm a safe distance from the left coronary artery. Ablation was delivered only after ensuring the catheter tip was ≥ 5 mm away from the coronary branches, and low-power, long-duration settings were applied to minimize the risk of coronary injury. Since the earliest activation site on the mapping was the RVOT, ablation was initiated there using the electrode catheter in the AIV region as an anatomical landmark (Figure 3a). Ablation was performed with low power and long duration (20 W for 120 s), which significantly reduced the PVCs; however, some PVCs persisted. Subsequently, additional ablation was attempted from the AS region, which was not the earliest site but was adjacent to the earliest site in the AIV region, using low-power settings (25 W for 65 s) (Figure 3b). Lastly, additional low-power, long-duration ablation with 30 W for 90 s applied from the LVOT region, again using the AIV catheter as an anatomical landmark (Figure 3c), suppressed the PVCs, and the procedure was ended. In total, radiofrequency applications consisted of 120 s at the RVOT, 65 s at the AS, and 90 s at the LVOT. At the RVOT, AS, and LVOT sites, the baseline impedance values ranged from 90 to 110 *Ω*, with a gradual impedance drop of approximately 10–15 *Ω* during each application, and no impedance rise was observed. The patient has remained free of recurrence following the catheter ablation. At 12 months of follow-up, no PVC recurrence was documented on standard 12-lead ECG and clinical visits. Although no repeat Holter monitoring was performed, the patient remained asymptomatic. A beta-blocker was continued, and no other antiarrhythmic drugs were prescribed.

## 3. Discussion

The significance of this case lies in two key aspects: first, the identification of the true earliest activation site using a microcatheter, and second, the application of prolonged low-power ablation applications from multiple regions surrounding this target site. This strategy was effective in this case in managing refractory OT-PVCs with a PBV2 and may represent a promising approach for similar patients.

OT-PVCs with a PBV2 are anatomically suggestive of an origin near the anterior interventricular groove. This area is bordered by the left anterior descending branch and the AIV, surrounded by thick epicardial fat, which often makes OT-PVCs originating from this region refractory to treatment. In the present report, 89% of non-PBV2 cases achieved long-term suppression postablation, while only about 58% of PBV2-related PVC ablation cases were amenable to long-term suppression [4]. One contributing factor to these poor outcomes is the difficulty in identifying the true earliest activation site, leading to missed ablation targets. In this case, the success was largely due to accurately identifying the earliest activation region using a microcatheter. Unlike previous reports describing long-duration ablation from adjacent chambers or successful ablation of AIV-origin PVCs from the RVOT [5, 6], our approach emphasized the systematic use of the AIV microcatheter as both an anatomical and electrophysiological landmark. However, even after locating the site, determining the optimal ablation strategy remains a future challenge. Nagashima et al. reported that for ventricular arrhythmias with an LV summit origin and earliest excitation in the distal great cardiac vein or AIV, cases treatable by ablation from the contralateral LVOT often exhibit an initial R wave in the V1 lead [7].

In the present case, an initial R wave was also observed in Lead V1, with the earliest activation in the AIV region. Anatomically, the AS was the closest structure to this earliest activation, while the fastest propagation site was in the RVOT among the three ablatable chambers (RVOT, AS, and LVOT). This discrepancy suggested that the critical ablation target lay in a region equidistant electrically and structurally from all three chambers. Thus, a low-power, prolonged ablation from the RVOT, AS, and LVOT, surrounding this earliest activation site, allowed for a deeper penetration into the energized area and better control of the PVCs. It has also been reported that the depth of lesions is deeper with a low-power, moderate-duration radiofrequency ablation than with a high-power, short-duration settings [8]. This approach proved effective in this case, where deeper lesion formation was required.

These results suggest that treatment of OT-PVCs with a PBV2 with prolonged ablation from multiple sites surrounding the earliest site may be a viable and effective treatment option in cases of OT-PVCs with a PBV2, and to summarize, we call this strategy “enveloped ablation” (Figure 3d). It should also be noted that in challenging cases where conventional unipolar ablation is insufficient, advanced techniques such as alcohol ablation or bipolar ablation have been reported as alternative strategies [9]. In our patient, however, careful modification of conventional unipolar ablation parameters was sufficient to achieve durable PVC suppression, and therefore, advanced techniques were not required.

## 4. Conclusions

We encountered a case in which detailed mapping-guided, prolonged low-power ablation from multiple sites effectively treated OT-PVCs with a PBV2. Although OT-PVCs with a PBV2 are typically refractory, our approach—termed “enveloped ablation”—involves targeted ablations at multiple sites surrounding the earliest activation site, highlighting its potential as an effective treatment strategy.

## Data Availability

The data that support the findings of this case report are available from the corresponding author upon reasonable request. The data are not publicly available due to privacy or ethical restrictions.
